# Association of Proton Pump Inhibitor Prophylaxis on Clinical Outcome in Acute Ischemic Stroke in China: A Multicenter Retrospective Cohort Study

**DOI:** 10.3390/jcm11236881

**Published:** 2022-11-22

**Authors:** Lei Fang, Wansi Zhong, Xiaoxian Gong, Zhicai Chen, Yi Chen, Shenqiang Yan, Min Lou

**Affiliations:** 1 Department of Neurology, The Second Affiliated Hospital of Zhejiang University, School of Medicine, Hangzhou 310009, China; 2 Department of Neurology, Zhejiang General Team Hospital of Chinese People’s Armed Police Force, Hangzhou 310051, China

**Keywords:** acute ischemic stroke, gastrointestinal bleeds, proton pump inhibitor prophylaxis, outcome

## Abstract

Background: Overtreatment with proton pump inhibitors (PPIs) in acute ischemic stroke (AIS) patients continues to grow. We aimed to investigate the frequency of PPI prophylaxis without an appropriate indication in AIS patients in China and clarify the association between PPI prophylaxis and long-term prognosis. Methods: Based on a multicenter stroke registry database, neurological outcomes, stroke events, recurrent ischemic strokes, and all-cause death were compared between patients with and without PPI prophylaxis. Results: A total of 4542 AIS were included, and 3335 (73.4%) received PPI prophylaxis. Patients with PPI prophylaxis were more likely to have a poor outcome at 1 year than those without PPI prophylaxis (33.3% vs. 25.8%, OR 1.321; 95% CI 1.102–1.584; *p* = 0.003). No significant differences were found in all-cause death, stroke event, or recurrent ischemic stroke at 1 year between the two groups. After propensity score matching, PPI prophylaxis was still independently associated with a higher rate of poor outcome (30.9% vs. 25.8%, OR 1.432; 95% CI 1.151–1.780; *p* = 0.001). Sensitivity analysis also showed that PPI prophylaxis increased the rate of a poor outcome in minor strokes or at different durations of PPI prophylaxis. Conclusions: Approximately 3/4 of AIS patients were given PPI prophylaxis during hospitalization, which was associated with a poor long-term outcome.

## 1. Introduction

China faces the greatest challenge from stroke in the world [1]. Recurrence is particularly challenging as the risk of recurrent stroke was estimated at 11.1% and 26.4% at first and five years after stroke onset, respectively [2]. To prevent recurrent stroke or stroke progression in acute ischemic stroke (AIS) patients, antiplatelet therapy is commonly used, with subsequent addition of proton pump inhibitors (PPIs) to prevent against potential gastrointestinal disease [3,4]. This is because randomized trials have shown that antiplatelets increase the risk of major gastrointestinal bleeds (GIB), while PPIs could reduce low-dose aspirin-associated gastrointestinal ulcers and bleeding by 70–90% [5]. 

Concerns have been raised about the adverse effects of PPI use. It was reported that, in the general population, PPI use alone increased the risk of a first-time ischemic stroke by 0.04% [6]. PPI use was also associated with an increased risk of cardiovascular events by 7.7% in aspirin-treated patients diagnosed with a first-time myocardial infarction [7]. 

PPI use still continues to grow every year in almost all western and eastern countries. It has been reported that 40% of patients used PPIs for a non-registered indication in the Netherlands, and more than 50% of patients failed to meet proper indications for PPI therapy in Colorado, calling into question the role of PPI misuse [8,9]. Indeed, PPIs prescribed without indication have been previously reported to range from 27% to 71% [10,11,12,13]. Therefore, we aimed to investigate the frequency of PPI prophylaxis in AIS patients and try to clarify the association of PPI prophylaxis with long-term prognosis among AIS patients in China.

## 2. Materials and Methods

### 2.1. Study Subjects

We retrospectively reviewed our prospectively collected database for consecutive patients with AIS who were admitted between December 2015 to December 2018 from 10 stroke centers. The data was from an automated enrollment computer-based online database of acute stroke patients for stroke management quality evaluation II (CASE II) registry (NCT 04487340), a longitudinal record of care for stroke inpatients. Patients aged ≥18 years and diagnosed with an AIS within 14 days of onset were included. We excluded patients who (1) had a pre-stroke modified Rankin scale (mRS) score ≥ 2, (2) had appropriate guideline-recommended indications for PPI use [14], and (3) had no follow-up mRS score at 1 year.

### 2.2. Ethics Statement

The Ethics Committee of the Second Affiliated Hospital of Zhejiang University, School of Medicine, approved the protocol. The clinical investigation was conducted according to the principles expressed in the Declaration of Helsinki. Thus, patient information was de-identified and anonymized and the informed consent requirement was waived by the Ethics Committee of the Second Affiliated Hospital of Zhejiang University, School of Medicine.

### 2.3. Clinical Data

Patient characteristics were recorded from the registry database, including demographic, clinical, and laboratory data including prescription of PPIs, age, gender, history of smoking, history of stroke, and comorbid conditions such as hypertension, diabetes, atrial fibrillation, hyperlipidemia, a baseline National Institutes of Health Stroke Scale (NIHSS) score, baseline systolic blood pressure (SBP), baseline diastolic blood pressure (DBP), blood glucose, and intravenous thrombolysis (IVT). Minor stroke was defined as a baseline NIHSS ≤ 5 [15]. PPI prophylaxis was defined as PPI use for AIS patients without appropriate indication during hospitalization. Non-PPI prophylaxis was defined as no PPI use during hospitalization. PPIs included omeprazole, pantoprazole, lansoprazole, esomeprazole, and rabeprazole.

All patients were followed up at 1 year by certified external clinical evaluators during a standardized telephone interview. All telephone interviews were recorded and traceable.

Clinical outcome was assessed with the mRS score and dichotomized into good outcome (mRS ≤ 2) and poor outcome (mRS > 2) at discharge and 1 year. Stroke events were defined as fatal or non-fatal acute events that fulfilled the typical symptoms of stroke (that is, people presenting clinical signs and symptoms suggestive of subarachnoid hemorrhage, intracerebral hemorrhage, or ischemic stroke) [16]. Recurrent ischemic stroke was defined as a new focal neurological deficit of vascular origin lasting > 24 h and without hemorrhage on computed tomography or MRI of the brain, which was included in the stroke event [17].

### 2.4. Statistical Analysis

Clinical characteristics were summarized as mean ± SD or median (25th–75th percentile) for quantitative variables and as proportions for categorical variables. The chi-square test was used to compare the dichotomous variables between groups, whereas for independent samples a two-tailed t-test or a Mann–Whitney U test was for the continuous variables. Variables with a *p*-value ≤ 0.05 in univariate analyses were entered into the binary logistic regression model. 

Propensity score-matched (PSM) analysis was used to minimize potential imbalances in the distribution of potential confounders between PPI prophylaxis and non-PPI prophylaxis. For matching, we used a structured, iterative propensity score model with the inclusion of age, history of smoking, hyperlipidemia, and the baseline NIHSS score to maximize the balance in the distribution of possible confounders between the two aforementioned groups. The corresponding propensity score was calculated for each subject, and a nearest-neighbor matching algorithm with a 1:1 allocation was subsequently implemented to match eligible patients with PPI prophylaxis and non-PPI prophylaxis. We used a conservative calliper size of 0.2 SDs of the logit of the PSM to provide adequate matching. Univariate analysis and binary logistic regression were repeated after propensity score matched analysis. To assess whether the results were influenced by the duration of PPI prophylaxis and baseline NIHSS, sensitivity analyses were performed by repeating the primary analysis between AIS patients with PPI prophylaxis lasting more than seven days and less than seven days (including non-PPI prophylaxis) and minor stroke patients. We also repeated the primary analysis after matching for all risk factors. All statistical analyses were performed using SPSS Version 22.0 (IBM, Armonk, NY, USA). A *p*-value < 0.05 was considered statistically significant. 

## 3. Results

**Figure 1:**
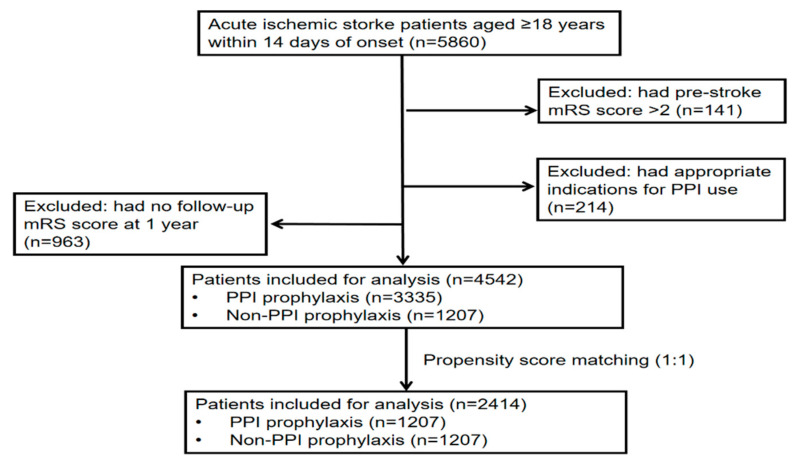
Flow chart of patient selection. mRS: modified Rankin scale; PPIs: proton pump inhibitors; Non-PPI: Non-proton pump inhibitors.

A total of 5860 AIS patients were included. As the flow chart of patient selection shows (Figure 1), the final analysis includes 4542 patients after excluding patients who had a pre-stroke mRS score > 2 (n = 141), had appropriate indications for PPI use (n = 214), and had no follow-up mRS score at 1 year (n = 963). Of the included patients, 3335 (73.4%) patients received PPI prophylaxis. The mean age was 67.3 ± 12.9 years, and 1311 (39.3) patients were women. The median NIHSS score was 3 (1–6). In total, 1561 (34.4%) achieved a poor outcome at discharge. During the 1-year follow-up, 296 (6.5%) patients suffered a stroke event, 289 (6.4%) patients had a recurrent ischemic stroke, and 1421 (31.3%) patients had a poor outcome.

### 3.1. Unmatched Analysis

As Table 1 shows, patients with PPI prophylaxis were younger, had a higher rate of smoking, and had a higher baseline NIHSS score than patients not receiving PPI prophylaxis. A higher rate of post-stroke pneumonia (14.0% vs. 8.1%, *p* < 0.001), gastrointestinal bleeding (1.5% vs. 0.1%, *p* < 0.001), poor outcome at discharge (36.4% vs. 28.8%, *p* < 0.001), and poor outcome at 1 year (33.3% vs. 25.8%, *p* < 0.001) was found among patients with PPI prophylaxis compared to non-PPI prophylaxis (Table 2). There were no significant differences in all-cause death, recurrent ischemic stroke, or stroke event at 1 year between the two groups (*p* >0.05). Binary logistic regression analysis revealed that PPI prophylaxis was associated with a higher rate of poor outcome at 1 year (OR 1.321; 95% CI 1.102–1.584; *p* = 0.003) (Table 2) but not with a higher rate of poor outcome at discharge (OR 1.052; 95% CI 0.880–1.257; *p* = 0.577) (Table S1). In addition, gastrointestinal bleeding was not significantly associated with poor outcome at 1 year (OR 1.554; 95% CI 0.730–3.305; *p* = 0.253). Post-stroke pneumonia was significantly associated with poorer outcomes (OR 2.285; 95% CI 2.015–3.308; *p* < 0.001).

### 3.2. Propensity-Matched Analysis

For the analysis of propensity score matching, we balanced baseline factors including age, history of smoking, hyperlipidemia, and baseline NIHSS score, resulting in 1207 patients with PPI prophylaxis and 1207 patients without PPI prophylaxis. 

Univariate and binary logistics models were repeated to identify independent predictors for poor outcome. Binary logistic regression analysis revealed that PPI prophylaxis was independently associated with a higher rate of poor outcome at 1 year (30.9% vs. 25.8%, OR 1.432; 95% CI 1.151–1.780; *p* = 0.001) (Table 1 and Table 2).

### 3.3. Sensitivity Analysis and Subgroup Analysis

A sensitivity analysis was conducted in minor stroke patients and AIS patients with different durations of PPI prophylaxis, showing that PPI prophylaxis was still independently associated with a poor outcome at 1 year (Table 3 and Tables S2–S4). After matching for age, history of smoking, hypertension, diabetes, atrial fibrillation, hyperlipidemia, and baseline NIHSS score, the results were consistent with the main analysis (Tables S5 and S6).

**Figure 2:**
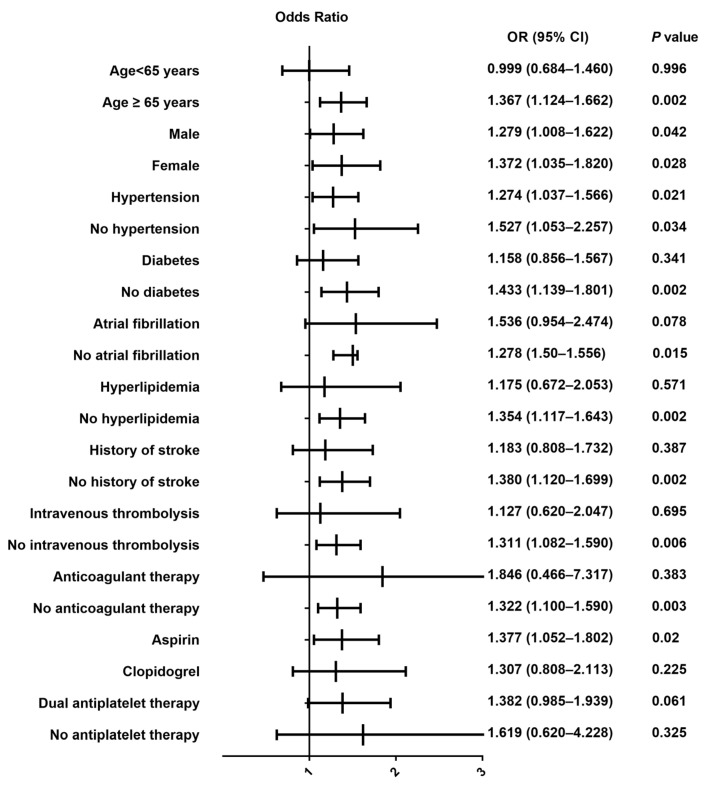
Forest plots for a poor outcome at one year in patients with different baseline characteristics.

Subgroup analysis (Figure 2) shows that the association between PPI prophylaxis and poor outcome at 1 year was not found in patients with an age < 65 years, diabetes, atrial fibrillation, hyperlipidemia, history of stroke, receiving intravenous thrombolysis, anticoagulant therapy, and non-aspirin user.

## 4. Discussion

In this study, the proportion of AIS patients receiving PPI prophylaxis was 73.4%. Both unmatched and propensity score analyses revealed that PPI prophylaxis increased the odds of a poor outcome one year after an AIS. 

The use of PPI in AIS patients should attract enough attention in China, as the rate of PPI prophylaxis in our study was higher than in previous studies (27–71%). Previous studies recommended the use of PPIs for stress ulcer prophylaxis in patients at high risk of gastrointestinal bleeding [18]. However, Mohammad et al. found that over 86% of patients in the general medical ward used PPIs inappropriately [19]. Ntaios et al. reported that 81.2% of hospitalized patients in an internal medicine department had no indications for the administration of PPIs, according to national guidelines [20]. The most common reasons for overuse of PPIs are the prevention of gastro-duodenal ulcers in patients without risk factors, stress ulcer prophylaxis in non-intensive care units, anticoagulant and antiplatelet treatment in patients without risk of gastric injury, and the overtreatment of functional dyspepsia [21]. In general, PPIs are perceived by clinicians as a harmless and relatively inexpensive preventative therapy for any digestive problem or as essential protection against possible drug-related gastric problems, eventually resulting in the overuse of PPIs in clinical practice. 

Evidence suggests that taking PPIs is associated with a small excess of cause-specific mortality, including death due to cardiovascular disease, chronic kidney disease, and upper gastrointestinal cancer [22]. Importantly, in the current study, PPI prophylaxis was also found to increase the rate of poor outcomes after one year. Even sensitivity analysis revealed such an association in minor stroke patients, excluding the possibility of confounding by indication. There are several potential mechanisms. (1) Long term exposure to PPIs increases oxidative stress, impairs endothelial function, and accelerates human endothelial senescence, which might lead to increased risk of cardiovascular morbidity and mortality [22,23]. (2) PPIs may inhibit the activity of nitric oxide (NO) synthase and facilitate the reduction of NO, while endothelium-derived NO is an important endogenous mediator of cerebral blood flow [24]. Plasma NO levels were significantly lower in stroke patients than in healthy volunteers, and studies have revealed that decreased plasma NO was associated with an unfavorable outcome in non-lacunar stroke patients [25]. (3) The application of PPIs could increase the risk of post-stroke pneumonia, which was associated with a poor long-term functional outcome [26,27]. Post-stroke pneumonia may be a contributor to the poor outcome in AIS patients with receiving prophylactic PPI. 

Guidelines recommend PPI prophylaxis in patients with a high risk of GIB [28]. We noticed that, from the results of subgroup analysis, most of the groups that did not reveal the association of PPI prophylaxis with poor outcome after one year were the ones that included patients who had a high risk of GIB. For example, it is commonly recognized that patients receiving antithrombotic agents experience an increased risk of GIB [29,30]. Blood glucose in patients with GIB was 0.8 mmol/L higher than that in patients without GIB. Patients with atrial fibrillation, hypertension, and a history of transient ischemic attack increased their risk of GIB by 8.5%, 5.3%, and 6.9%, respectively [31,32,33,34,35]. It was reported that GIB after IVT occurred in 5.3% of patients [36]. Therefore, our finding strongly supports that PPI prophylaxis should be appropriately used in patients with a high risk of GIB in clinical practice rather than all AIS patients, as guidelines for PPI prophylaxis recommend. 

This is the first study to explore the impact of PPI prophylaxis on the long-term neurological outcome of AIS. Our results suggested that the inappropriate use of PPIs should be discouraged in order to decrease the potential for poor outcomes, which have public health implications, considering the high prevalence of PPI prophylaxis. Our findings also highlight the importance of risk evaluation for GIB before PPI prophylaxis. 

Our study has several limitations. First, our study had a retrospective design and a potential risk of selection bias, although we prospectively collected data using a multicenter stroke registry, and we have attempted to control for confounding using propensity score matched analysis to reduce the biases of the results. Second, the duration of PPIs and the usage of PPIs after discharge were not recorded exactly, which may have a different effect on outcome, though we have conducted a sensitivity analysis in AIS patients with PPI prophylaxis for more than seven days and less than seve days (including non-PPI prophylaxis). Third, we did not evaluate the impact of different types of PPIs. It is not clear whether the pharmacokinetics and pharmacodynamics of PPIs would change their underlying mechanisms. Finally, although almost 75% of patients in this study had a minor stroke, data on treatment in an intensive care unit and controlled mechanical ventilation were not available, which may influence the stroke outcome.

## 5. Conclusions

PPI prophylaxis in hospitalized AIS patients was associated with higher rates of poor long-term outcomes. Rigorous assessments of PPI prophylaxis for those with a high risk of major gastrointestinal bleeds are warranted.

## Figures and Tables

**Table 1:** Demographic and clinical characteristics of the study population.

Variables	Unmatched		*p* Value	Propensity-Matched		*p* Value
	PPI Prophylaxis (n = 3335)	Non-PPI Prophylaxis (n = 1207)		PPI Prophylaxis (n = 1207)	Non-PPI Prophylaxis (n = 1207)	
Age, years	67.3 ± 12.9	68.3 ± 12.3	0.020	68.2 ± 12.5	68.3 ± 12.3	0.763
Female	1311 (39.3)	472 (39.1)	0.918	501 (41.5)	472 (39.1)	0.245
**Risk factors**						
History of smoking	1244 (37.3)	411 (34.1)	0.047	403 (33.4)	411 (34.1)	0.763
History of stroke	609 (18.3)	227 (18.8)	0.697	218 (18.1)	227 (18.8)	0.675
Hypertension	2489(74.6)	907 (75.1)	0.757	910 (75.4)	907 (75.1)	0.925
Diabetes mellitus	1027 (30.8)	370 (30.7)	0.942	345 (28.6)	370 (30.7)	0.285
Atrial fibrillation	483 (14.5)	159 (13.2)	0.268	156 (12.9)	159 (13.2)	0.904
Hyperlipidemia	335 (10.0)	155 (12.8)	0.008	150 (12.4)	155 (12.8)	0.806
**Clinical variables**						
Baseline NIHSS	3 (1–6)	2 (1–4)	<0.001	2 (1–4)	2 (1–4)	0.789
Baseline SBP, mmHg	150.5 ± 22.9	150.8 ± 23.1	0.770	150.8 ± 23.0	150.8 ± 23.1	0.950
Baseline DBP, mmHg	84.2 ± 13.7	84.8 ± 26.2	0.271	84.1 ± 13.6	84.2 ± 13.4	0.921
Blood glucose, mmol/L	6.00 ± 2.17	6.12 ± 2.33	0.124	5.84 ± 2.00	6.12 ± 2.33	0.002
Intravenous thrombolysis	286 (8.6)	100 (8.3)	0.810	71 (5.9)	100 (8.3)	0.026
**Outcome**						
Poor outcome at discharge	1213 (36.4)	348 (28.8)	<0.001	370 (30.7)	348 (28.8)	0.350
Poor outcome at 1 year	1109 (33.3)	312 (25.8)	<0.001	373 (30.9)	312 (25.8)	0.007
All-cause death	359 (10.8)	106 (8.8)	0.053	117 (9.7)	106 (8.8)	0.482
Stroke event	220 (7.4)	76 (6.9)	0.634	86 (7.9)	76 (6.9)	0.414
Recurrent ischemic stroke	213 (7.2)	76 (6.9)	0.837	81 (7.4)	76 (6.9)	0.679
Gastrointestinal bleeding	51 (1.5)	1 (0.1)	<0.001	19 (1.6)	1 (0.1)	<0.001
Post-stroke pneumonia	466 (14.0)	98 (8.1)	<0.001	137 (11.4)	98 (8.1)	0.009

PPIs: proton pump inhibitors, NIHSS: national institutes of health stroke scale, SBP: systolic blood pressure, DBP: diastolic blood pressure.

**Table 2:** Univariate comparison and multivariate analysis for poor outcome at one year in unmatched and propensity-matched patients.

Variables	Unmatched					Propensity-Matched				
	Univariate Analysis			Multivariate Analysis		Univariate Analysis			Multivariate Analysis	
	Poor Outcome (n = 1421)	Good Outcome (n = 3121)	*p* Value	OR (95% CI)	*p* Value	Poor Outcome (n = 685)	Good Outcome (n = 1729)	*p* Value	OR (95% CI)	*p* Value
Age, years	75.0 ± 10.9	64.3 ± 12.1	<0.001	1.091 (1.082–1.100)	<0.001	75.9 ± 10.5	65.2 ± 11.8	<0.001	1.095 (1.083–1.108)	<0.001
Female	673 (47.4)	1110 (35.6)	<0.001	1.293 (1.068–1.566)	0.008	325 (47.4)	648 (37.5)	<0.001	1.172 (0.904–1.518)	0.232
**Risk factors**										
History of smoking	421 (29.6)	1234 (39.5)	<0.001	1.151 (0.940–1.410)	0.174	184 (26.9)	630 (36.4)	<0.001	1.120 (0.843–1.489)	0.435
History of stroke	396 (27.9)	440 (14.1)	<0.001	2.052 (1.699–2.478)	<0.001	188 (27.4)	257 (14.9)	<0.001	1.771 (1.368–2.293)	<0.001
Hypertension	1126 (79.2)	2270 (72.7)	<0.001	1.126 (0.929–1.364)	0.227	559 (81.6)	1258 (72.8)	<0.001	1.317 (1.002–1.730)	0.049
Diabetes mellitus	491 (34.6)	906 (29.0)	0.001	1.658 (1.400–1.964)	<0.001	234 (34.2)	481 (27.8)	0.003	1.847 (1.458–2.339)	<0.001
Atrial fibrillation	368 (25.9)	274 (8.8)	<0.001	1.155 (0.923–1.447)	0.208	152 (22.2)	163 (9.4)	<0.001	1.139 (0.836–1.553)	0.410
Hyperlipidemia	110 (7.7)	380 (12.2)	<0.001	0.862 (0.660–1.126)	0.277	68 (9.9)	237 (13.7)	0.012	0.922 (0.656–1.296)	0.639
**Clinical variables**										
Baseline NIHSS	6 (3–11)	2 (1–4)	<0.001	1.260 (1.235–1.285)	<0.001	4 (2–9)	2 (1–3)	<0.001	1.295 (1.253–1.338)	<0.001
Baseline SBP, mmHg	152.5 ± 23.7	149.7 ± 22.6	<0.001	NA	NA	153.0 ± 23.2	149.8 ± 22.9	0.003	NA	NA
Baseline DBP, mmHg	82.4 ± 13.5	85.0 ± 13.5	<0.001	NA	NA	82.9 ± 13.3	84.7 ± 13.5	0.003	NA	NA
Blood glucose, mmol/L	6.43 ± 2.56	5.85 ± 2.01	<0.001	NA	NA	6.28 ± 2.47	5.86 ± 2.03	<0.001	NA	NA
Intravenous thrombolysis	138 (9.7)	248 (7.9)	0.051	NA	NA	50 (7.3)	121 (7.0)	0.792	NA	NA
PPI prophylaxis	1109 (78.0)	2226 (71.3)	<0.001	1.321 (1.102–1.584)	0.003	373 (54.5)	834 (48.2)	0.007	1.432 (1.151–1.780)	0.001

Values are mean (SD), median (interquartile range), or No. (%) as appropriate. PPIs: proton pump inhibitors, NIHSS: national institutes of health stroke scale, SBP: systolic blood pressure, DBP: Diastolic blood pressure.

**Table 3:** Univariate comparison and multivariate analysis for poor outcome at one year between AIS patients with PPI prophylaxis more than seven days and less than seven days.

Variables	Univariate Analysis			Multivariate Analysis	
	Poor Outcome (n = 1421)	Good Outcome (n = 3121)	*p* Value	OR (95% CI)	*p* Value
Age, years	75.0 ± 10.9	64.3 ± 12.1	<0.001	1.091 (1.082–1.100)	<0.001
Female, n (%)	673 (47.4)	1110 (35.6)	<0.001	1.298 (1.072–1.571)	0.007
**Risk factors**					
History of smoking	421 (29.6)	1234 (39.5)	<0.001	1.153 (0.940–1.410)	0.170
History of stroke	396 (27.9)	440 (14.1)	<0.001	2.046 (1.695–2.470)	<0.001
Hypertension	1126 (79.2)	2270 (72.7)	<0.001	1.130 (0.932–1.369)	0.213
Diabetes mellitus	491 (34.6)	906 (29.0)	0.001	1.656 (1.399–1.962)	<0.001
Atrial fibrillation	368 (25.9)	274 (8.8)	<0.001	1.161 (0.928–1.454)	0.192
Hyperlipidemia	110 (7.7)	380 (12.2)	<0.001	0.849 (0.650–1.109)	0.231
**Clinical variables**					
Baseline NIHSS	6 (3–11)	2 (1–4)	<0.001	1.260 (1.234–1.285)	<0.001
Baseline SBP, mmHg	152.5 ± 23.7	149.7 ± 22.6	<0.001	NA	NA
Baseline DBP, mmHg	82.4 ± 13.5	85.0 ± 13.5	<0.001	NA	NA
Blood glucose, mmol/L	6.43 ± 2.56	5.85 ± 2.01	<0.001	NA	NA
Intravenous thrombolysis	138 (9.7)	248 (7.9)	0.051	NA	NA
PPI prophylaxis	890 (62.6)	1707 (54.7)	<0.001	1.181 (1.006–1.388)	0.042

Values are mean (SD), median (interquartile range), or No. (%) as appropriate. PPIs: proton pump inhibitors, NIHSS: national institutes of health stroke scale, SBP: systolic blood pressure, DBP: diastolic blood pressure.

## Data Availability

The raw data supporting the conclusions of this article will be made available by the authors on reasonable request, without undue reservation (contact via zhejiangkeyan@163.com).

## Supplementary Materials

The following supporting information can be downloaded at: https://www.mdpi.com/article/10.3390/jcm11236881/s1, Table S1: Univariate comparison and multivariate analysis for poor outcome at discharge in unmatched and propensity-matched patients; Table S2: Demographic and clinical characteristics of the study population for long-term outcomes between AIS patients with PPI prophylaxis more than seven days and less than seven days; Table S3: Demographic and clinical characteristics of minor acute ischemic stroke; Table S4: Univariate comparison and multivariate analysis for poor outcome after one year in unmatched and propensity-matched minor acute ischemic stroke; Table S5: Demographic and Clinical Characteristics of Study Population (Matching for age, history of smoking, hypertension, diabetes, atrial fibrillation, hyperlipidemia, and baseline NIHSS score); Table S6: Univariate comparison and multivariate analysis for poor outcome at 1 year in unmatched and propensi-ty-matched patients (Matching for age, history of smoking, hypertension, diabetes, atrial fibrillation, hyperlipidemia, and baseline NIHSS score).
