# Supplementary material for: Association of Proton Pump Inhibitor Prophylaxis on Clinical Outcome in Acute Ischemic Stroke in China: A Multicenter Retrospective Cohort Study
Source: J Clin Med. 2022 Nov 22;11(23):6881. doi: 10.3390/jcm11236881 (PMC9740641; doi:10.3390/jcm11236881)
Supplement: Supplementary file 1 [file jcm-11-06881-s001.zip › jcm-2017311-supplementary.pdf]

Table S1. Univariate comparison and multivariate analysis for poor outcome at discharge in unmatched and propensity-matched patients

Variables	Unmatched					Propensity-matched				
	Univariate analysis			Multivariate analysis		Univariate analysis			Multivariate analysis	
	Poor outcome (n=1561)	Good outcome (n=2981)	*P* value	OR (95%CI)	*P* value	Poor outcome (n=718)	Good outcome (n=1696)	*P* value	OR (95%CI)	*P* value
Age, years	70.2±12.5	66.2±12.6	<0.001	1.013 (1.006-1.019)	<0.001	71.1±12.2	67.1±12.3	<0.001	1.016 (1.006-1.026)	0.001
Female	687 (44.0)	1096 (36.8)	<0.001	1.022 (0.844-1.238)	0.824	322 (44.8)	651 (38.4)	0.003	0.885 (0.679-1.153)	0.366
**Risk factors**										
History of smoking	505 (32.4)	1150 (38.6)	<0.001	0.891 (0.730-1.087)	0.254	198 (27.6)	616 (36.3)	<0.001	0.696 (0.522-0.928)	0.014
History of stroke	341 (21.8)	495 (16.6)	<0.001	1.116 (0.917-1.359)	0.273	163 (22.7)	282 (16.6)	0.001	1.147 (0.870-1.513)	0.329
Hypertension	1185 (75.9)	2211 (74.2)	0.208	NA	NA	556 (77.4)	1261 (74.4)	0.110	NA	NA
Diabetes mellitus	513 (32.9)	884 (29.7)	0.028	1.302 (1.103-1.537)	0.002	223 (31.1)	492 (29.0)	0.329	NA	NA
Atrial fibrillation	363 (23.3)	279 (9.4)	<0.001	1.232 (0.964-1.574)	0.095	141 (19.6)	174 (10.3)	<0.001	0.915 (0.646-1.296)	0.616
Hyperlipidemia	130 (8.3)	360 (12.1)	<0.001	0.791 (0.610-1.025)	0.076	77 (10.7)	228 (13.4)	0.070	NA	NA
**Clinical variables**										
Baseline NIHSS	7 (4-12)	2 (1-3)	<0.001	1.489 (1.449-1.531)	<0.001	6 (3-10)	2 (1-3)	<0.001	1.543 (1.477-1.611)	<0.001
Baseline SBP, mmHg	152.6±23.9	149.5±22.4	<0.001	1.004 (1.001-1.007)	0.022	153.6±23.7	149.6±22.6	<0.001	1.003 (0.998-1.008)	0.276
Baseline DBP, mmHg	83.8±13.5	84.4±13.5	0.124	NA	NA	84.4±13.6	84.0±13.4	0.525	NA	NA
Blood glucose, mmol/L	6.43±2.52	5.82±1.99	<0.001	NA	NA	6.38±2.51	5.80±1.98	<0.001	1.107 (1.053-1.163)	<0.001
Intravenous thrombolysis	157 (10.1)	229 (7.7)	0.007	0.434 (0.316-0.597)	<0.001	56 (7.8)	115 (6.8)	0.386	NA	NA
PPI prophylaxis	1213 (77.7)	2122 (71.2)	<0.001	1.052 (0.880-1.257)	0.577	370 (51.5)	837 (49.4)	0.350	1.124 (0.899-1.405)	0.305

Value are mean (SD), median (interquartile range), or No. (%) as appropriate. PPIs: Proton Pump Inhibitors, NIHSS: National Institutes of Health Stroke Scale, SBP: Systolic Blood Pressure, DBP: Diastolic Blood Pressure.

Table S2. Demographic and Clinical Characteristics of Study Population for long-term outcome between AIS patients with PPI prophylaxis more than 7 days and less than 7 days.

**Variables**	**PPI prophylaxis (n=2597)**	**Non-PPI prophylaxis (n=1945)**	***P* value**
Age, years	67.5±12.8	67.7±12.6	0.562
Female	1010 (38.9)	773 (39.7)	0.580
**Risk factors**			
History of smoking	981 (37.8)	674 (34.7)	0.032
History of stroke	474 (18.3)	362 (18.6)	0.757
Hypertension	1918 (73.9)	1478 (76.0)	0.105
Diabetes mellitus	805 (31.0)	592 (30.4)	0.697
Atrial fibrillation	377 (14.5)	265 (13.6)	0.414
Hyperlipidemia	260 (10.0)	230 (11.8)	0.053
**Clinical variables**			
Baseline NIHSS	3 (1-7)	2 (1-4)	<0.001
Baseline SBP, mmHg	150.5±22.7	150.7±23.4	0.693
Baseline DBP, mmHg	84.2±13.4	84.3±13.7	0.767
Blood glucose, mmol/L	6.02±2.20	6.06±2.23	0.515
Intravenous thrombolysis	205 (7.9)	181 (9.3)	0.096
**Outcome**			
Poor outcome at discharge	1006 (38.7)	555 (28.5)	<0.001
Poor outcome at 1 year	890 (34.3)	531 (27.3)	<0.001
All-cause death	252 (9.7)	213 (11.0)	0.182
Stroke event	170 (7.2)	126 (7.3)	1.000
Recurrent ischemic stroke	167 (7.1)	122 (7.0)	0.951

Value are mean (SD), median (interquartile range), or No. (%) as appropriate. PPIs: Proton Pump Inhibitors, NIHSS: National Institutes of Health Stroke Scale, SBP: Systolic Blood Pressure, DBP: Diastolic Blood Pressure.

Table S3. Demographic and Clinical Characteristics of minor acute ischemic stroke

Variables	Unmatched		*P* value	Propensity-matched		*P* value
	PPI prophylaxis (n=2411)	Non-PPI prophylaxis (n=978)		PPI prophylaxis (n=978)	Non-PPI prophylaxis (n=978)	
Age, years	66.4±12.8	67.3±12.2	0.056	67.9±12.6	67.3±12.2	0.253
Female	894 (37.1)	368 (37.6)	0.784	384 (39.3)	368 (37.6)	0.486
**Risk factors**						
History of smoking	938 (38.9)	345 (35.3)	0.051	336 (34.4)	345 (35.3)	0.704
History of stroke	411 (17.0)	169 (17.3)	0.880	161 (16.5)	169 (17.3)	0.673
Hypertension	1801 (74.7)	728 (74.4)	0.896	723 (73.9)	728 (74.4)	0.836
Diabetes mellitus	752 (31.2)	300 (30.7)	0.774	315 (32.2)	300 (30.7)	0.495
Atrial fibrillation	223 (9.2)	102 (10.4)	0.303	82 (8.4)	102 (10.4)	0.141
Hyperlipidemia	272 (11.3)	126 (12.9)	0.195	121 (12.4)	126 (12.9)	0.785
**Clinical variables**						
Baseline NIHSS	2 (1-3)	2 (1-3)	<0.001	2 (1-3)	2 (1-3)	0.472
Baseline SBP, mmHg	150.1±22.6	149.5±23.2	0.501	150.3±22.6	149.5±23.2	0.417
Baseline DBP, mmHg	84.6±13.4	83.9±13.3	0.207	83.9±13.4	83.9±13.3	0.984
Blood glucose, mmol/L	5.86±2.04	5.99±2.12	0.094	5.87±2.06	5.99±2.12	0.206
Intravenous thrombolysis	155 (6.4)	61 (6.2)	0.877	58 (5.9)	61 (6.2)	0.850
**Outcome**						
Poor outcome at discharge	439 (18.2)	172 (17.6)	0.693	176 (18.0)	172 (17.6)	0.859
Poor outcome at 1 year	492 (20.4)	171 (17.5)	0.056	234 (23.9)	171 (17.5)	0.001
All-cause death	127 (5.3)	47 (4.8)	0.607	61 (6.2)	47 (4.8)	0.198
Stroke event	174 (7.6)	63 (6.8)	0.457	79 (8.6)	63 (6.8)	0.139
Recurrent ischemic stroke	170 (7.4)	63 (6.8)	0.549	77 (8.4)	63 (6.8)	0.189

Value are mean (SD), median (interquartile range), or No. (%) as appropriate. PPIs: Proton Pump Inhibitors, NIHSS: National Institutes of Health Stroke Scale, SBP: Systolic Blood Pressure, DBP: Diastolic Blood Pressure.

Table S4. Univariate comparison and multivariate analysis for poor outcome at 1 year in unmatched and propensity-matched minor acute ischemic stroke.

Variables	Unmatched					Propensity-matched				
	Univariate analysis			Multivariate analysis		Univariate analysis			Multivariate analysis	
	Poor outcome (n=663)	Good outcome (n=2726)	*P* value	OR (95%CI)	*P* value	Poor outcome (n=405)	Good outcome (n=1551)	*P* value	OR (95%CI)	*P* value
Age,years	76.0±10.7	64.4±12.0	<0.001	1.103 (1.091-1.115)	<0.001	76.4±10.2	65.3±11.9	<0.001	1.102 (1.087-1.119)	<0.001
Female	290 (43.7)	972 (35.7)	<0.001	1.213 (0.958-1.535)	0.109	188 (46.4)	564 (36.4)	<0.001	1.491 (1.089-2.042)	0.013
**Risk factors**										
History of smoking	421 (29.6)	1076 (39.5)	<0.001	1.144 (0.889-1.471)	0.295	112 (27.7)	569 (36.7)	0.001	1.181 (0.836-1.669)	0.344
History of stroke	190 (28.7)	390 (14.3)	<0.001	1.928 (1.528-2.432)	<0.001	122 (30.1)	208 (13.4)	<0.001	2.347 (1.732-3.181)	<0.001
Hypertension	531 (80.1)	1998 (73.3)	<0.001	1.030 (0.804-1.319)	0.815	321 (79.3)	1130 (72.9)	0.009	0.971 (0.704-1.340)	0.859
Diabetes mellitus	258 (38.9)	794 (29.1)	<0.001	1.536 (1.191-1.981)	0.001	162 (40.0)	453 (29.2)	0.003	1.606 (1.147-2.249)	0.006
Atrial fibrillation	111 (16.7)	214 (7.9)	<0.001	1.356 (1.020-1.803)	0.036	58 (14.3)	126 (8.1)	<0.001	1.336 (0.903-1.976)	0.146
Hyperlipidemia	64 (9.7)	334 (12.3)	0.069	NA	NA	46 (11.4)	201 (13.0)	0.450	NA	NA
**Clinical variables**										
Baseline NIHSS	2 (1-4)	2 (1-3)	<0.001	1.276 (1.195-1.362)	<0.001	2 (1-4)	2 (1-3)	<0.001	1.306 (1.197-1.425)	<0.001
Baseline SBP, mmHg	151.3±23.2	149.6±22.7	0.075	NA	NA	150.7±23.4	149.7±22.7	0.433	NA	NA
Baseline DBP, mmHg	81.8±12.6	85.0±13.5	<0.001	0.999 (0.991-1.007)	0.856	81.2±12.4	84.7±13.5	<0.001	0.996 (0.986-1.007)	0.452
Blood glucose, mmol/L	6.15±2.37	5.83±1.98	0.001	1.079 (1.020-1.142)	0.008	6.19±2.37	5.86±2.01	0.007	1.007 (0.998-1.161)	0.055
Intravenous thrombolysis	38 (5.7)	178 (6.5)	0.480	NA	NA	22 (5.4)	97 (6.3)	0.640	NA	NA
PPI prophylaxis	492 (74.2)	1919 (70.4)	0.056	1.315 (1.052-1.645)	0.016	234 (57.8)	744 (48.0)	0.001	1.569 (1.210-2.033)	0.001

Value are mean (SD), median (interquartile range), or No. (%) as appropriate. PPIs: Proton Pump Inhibitors, NIHSS: National Institutes of Health Stroke Scale, SBP: Systolic Blood Pressure, DBP: Diastolic Blood Pressure.

Table S5. Demographic and Clinical Characteristics of Study Population (Matching for age, history of smoking, hypertension, diabetes, atrial fibrillation, hyperlipidemia, and baseline NIHSS score)

Variables	Unmatched		P value	Propensity-matched		P value
	PPI prophylaxis (n=3335)	Non-PPI prophylaxis (n=1207)		PPI prophylaxis (n=1207)	Non-PPI prophylaxis (n=1207)	
Age, years	67.3±12.9	68.3±12.3	0.020	68.4±12.6	68.3±12.3	0.909
Female	1311 (39.3)	472 (39.1)	0.918	486 (40.3)	472 (39.1)	0.589
**Risk factors**						
History of smoking	1244 (37.3)	411 (34.1)	0.047	415 (34.4)	411 (34.1)	0.898
History of stroke	609 (18.3)	227 (18.8)	0.697	215 (17.8)	227 (18.8)	0.563
Hypertension	2489(74.6)	907 (75.1)	0.757	914 (75.7)	907 (75.1)	0.777
Diabetes mellitus	1027 (30.8)	370 (30.7)	0.942	326 (27.0)	370 (30.7)	0.053
Atrial fibrillation	483 (14.5)	159 (13.2)	0.268	149 (12.3)	159 (13.2)	0.583
Hyperlipidemia	335 (10.0)	155 (12.8)	0.008	145 (12.0)	155 (12.8)	0.579
**Clinical variables**						
Baseline NIHSS	3 (1-6)	2 (1-4)	<0.001	2 (1-4)	2 (1-4)	0.822
Baseline SBP, mmHg	150.5±22.9	150.8±23.1	0.770	151.1±22.7	150.8±23.1	0.685
Baseline DBP, mmHg	84.2±13.7	84.8±26.2	0.271	84.2±13.8	84.2±13.4	0.924
Blood glucose, mmol/L	6.00±2.17	6.12±2.33	0.124	5.76±1.84	6.12±2.33	<0.001
Intravenous thrombolysis	286 (8.6)	100 (8.3)	0.810	101 (8.4)	100 (8.3)	1.000
**Outcome**						
Poor outcome at discharge	1213 (36.4)	348 (28.8)	<0.001	371 (30.7)	348 (28.8)	0.328
Poor outcome at 1 year	1109 (33.3)	312 (25.8)	<0.001	367 (30.4)	312 (25.8)	0.014
All-cause death	359 (10.8)	106 (8.8)	0.053	104 (8.6)	106 (8.8)	0.942

Stroke event	220 (7.4)	76 (6.9)	0.634	88 (8.0)	76 (6.9)	0.372
Recurrent ischemic stroke	213 (7.2)	76 (6.9)	0.837	83 (7.5)	76 (6.9)	0.621
Gastrointestinal bleeding	51 (1.5)	1 (0.1)	<0.001	18 (1.5)	1 (0.1)	<0.001
Post-stroke pneumonia	466 (14.0)	98 (8.1)	<0.001	141 (11.7)	8 (8.1)	0.004

Value are mean (SD), median (interquartile range), or No. (%) as appropriate. PPIs: Proton Pump Inhibitors, NIHSS: National Institutes of Health Stroke Scale, SBP: Systolic Blood Pressure, DBP: Diastolic Blood Pressure.

Table S6. Univariate comparison and multivariate analysis for poor outcome at 1 year in unmatched and propensi-ty-matched patients (Matching for age, history of smoking, hypertension, diabetes, atrial fibrillation, hyperlipidemia, and baseline NIHSS score)

Variables	Unmatched					Propensity-matched				
	Univariate analysis			Multivariate analysis		Univariate analysis			Multivariate analysis	
	Poor outcome (n=1421)	Good outcome (n=3121)	*P* value	OR (95%CI)	*P* value	Poor outcome (n=679)	Good outcome (n=1735)	*P* value	OR (95%CI)	*P* value
Age, years	75.0±10.9	64.3±12.1	<0.001	1.091 (1.082-1.100)	<0.001	76.3±9.9	65.3±12.0	<0.001	1.100 (1.087-1.113)	<0.001
Female	673 (47.4)	1110 (35.6)	<0.001	1.293 (1.068-1.566)	0.008	313 (46.1)	645 (37.2)	<0.001	1.128 (0.868-1.465)	0.368
**Risk factors**										
History of smoking	421 (29.6)	1234 (39.5)	<0.001	1.151 (0.940-1.410)	0.174	184 (27.1)	642 (37.0)	<0.001	1.056 (0.795-1.403)	0.708
History of stroke	396 (27.9)	440 (14.1)	<0.001	2.052 (1.699-2.478)	<0.001	196 (28.9)	246 (14.2)	<0.001	2.103 (1.620-2.729)	<0.001
Hypertension	1126 (79.2)	2270 (72.7)	<0.001	1.126 (0.929-1.364)	0.227	555 (81.7)	1266 (73.0)	<0.001	1.230 (0.933-1.622)	0.141
Diabetes mellitus	491 (34.6)	906 (29.0)	0.001	1.658 (1.400-1.964)	<0.001	221 (32.5)	475 (27.4)	0.012	1.777 (1.394-2.266)	<0.001
Atrial fibrillation	368 (25.9)	274 (8.8)	<0.001	1.155 (0.923-1.447)	0.208	153 (22.5)	155 (8.9)	<0.001	1.172 (0.853-1.609)	0.328
Hyperlipidemia	110 (7.7)	380 (12.2)	<0.001	0.862 (0.660-1.126)	0.277	68 (10.0)	232 (13.4)	0.028	1.061 (0.753-1.495)	0.735
**Clinical variables**										
Baseline NIHSS	6(3-11)	2(1-4)	<0.001	1.260 (1.235-1.285)	<0.001	4 (2-9)	2 (1-3)	<0.001	1.297 (1.255-1.341)	<0.001
Baseline SBP, mmHg	152.5±23.7	149.7±22.6	<0.001	NA	NA	153.5±23.0	149.9±22.8	0.001	NA	NA
Baseline DBP, mmHg	82.4±13.5	85.0±13.5	<0.001	NA	NA	82.7±13.4	84.8±13.6	<0.001	NA	NA
Blood glucose, mmol/L	6.43±2.56	5.85±2.01	<0.001	NA	NA	6.29±2.52	5.80±1.90	<0.001	NA	NA
Intravenous thrombolysis	138 (9.7)	248 (7.9)	0.051	NA	NA	63 (9.3)	138 (8.0)	0.288	NA	NA
PPI prophylaxis	1109 (78.0)	2226 (71.3)	<0.001	1.321 (1.102-1.584)	0.003	367 (54.1)	840 (48.4)	0.014	1.409 (1.130-1.758)	0.002

Value are mean (SD), median (interquartile range), or No. (%) as appropriate. PPIs: Proton Pump Inhibitors, NIHSS: National Institutes of Health Stroke Scale, SBP: Systolic Blood Pressure, DBP: Diastolic Blood Pressure.
